# Genomic analysis of the *SMN1* gene region in patients with clinically diagnosed spinal muscular atrophy: a retrospective observational study

**DOI:** 10.1186/s13023-025-03568-9

**Published:** 2025-02-07

**Authors:** Tamaki Kato, Mamoru Yokomura, Yutaka Osawa, Kensuke Matsuo, Yuji Kubo, Taihei Homma, Kayoko Saito

**Affiliations:** 1 Institute of Medical Genetics, Tokyo Women’s Medical University, 8-1 Kawada-cho, Shinjuku-ku, Tokyo, 162-8666 Japan; 2 Department of Neurology, Kawasaki Medical School, Kurashiki, Okayama Japan; 3 Division of Pediatrics, Kyoto Tanabe Central Hospital, Kyoto, Japan; 4 Biogen Japan Ltd., Tokyo, Japan

**Keywords:** Genomics, Spinal muscular atrophy, Mutation, Long-range PCR, Next-generation sequencing, Single nucleotide variant, *SMN1* gene, *SMN2* gene

## Abstract

**Background:**

Spinal muscular atrophy (SMA) is an autosomal recessive neuromuscular disease. Most patients with SMA have a mutation in the *survival motor neuron 1* (*SMN1*) gene on chromosome 5q. With current genetic testing, *SMN1* copy number is determined; a diagnosis is reached when the copy number is zero. When the *SMN1* copy number is 1, exons and intron/exon boundaries of the allele are examined for single-nucleotide variants (SNVs). Genetically undiagnosed cases of SMA exist when 2 copies of *SMN1* exist or when a SNV is in the deep intron. Furthermore, *SMN1* is highly homologous to *SMN2*; therefore, it is expected that many SNVs have not been elucidated.

**Methods:**

This retrospective observational study conducted in Japan used pre-collected DNA samples from patients with clinically diagnosed SMA. Enrollment period was January 28, 2020 to September 30, 2021. SNV analysis of *SMN1* (exon 1–8 and intron 1–7) was conducted by long-range polymerase chain reaction and next-generation sequencing.

**Results:**

From 336 DNA samples collected from patients, 62 patient samples were included in the SNV analysis. Two patients have been genetically diagnosed (a heterozygous variant in intron 6 with 1 copy of *SMN1*; a homozygous missense mutation in exon 3 with 2 copies of *SMN1*). Three SNVs in intron 6, c.834+1506A>G (n = 9), c.834+1751G>A (n = 2), and c.835-367C>A (n = 5) were identified; all were numerically, and c.834+1506A>G and c.835-367C>A were significantly, more frequent in patients with 0 copies versus those with ≥ 1 copy of exon 7 in *SMN1*. We confirmed 3 hybrid *SMN* gene types in 5 patients that contained *SMN2* gene sequence (aaTgg) flanked by upstream “t” and downstream “G” *SMN1* sequence.

**Conclusions:**

In this study of patients with clinically diagnosed SMA, 2 cases with genetic *SMN* types were identified that would not have been identified through current genetic testing, which examines *SMN1* deletions only. Furthermore, for 1 patient with a homozygous *SMN1* missense mutation, SMA was not suspected by the current copy number screening method. This study demonstrated the importance of performing full-length sequencing for clinically diagnosed SMA to complement current screening methods.

*Trial registration*: University Hospital Medical Information Network Clinical Trials Registry (Number: UMIN000040095).

**Supplementary Information:**

The online version contains supplementary material available at 10.1186/s13023-025-03568-9.

## Introduction

Spinal muscular atrophy (SMA) is an autosomal recessive neuromuscular disease characterized by muscle weakness and atrophy, resulting from progressive degeneration of anterior horn cells in the spinal cord [1]. SMA is one of the leading genetic causes of infant death, with an estimated global incidence (birth prevalence) of approximately 1 in 10,000 live births [2–5], and in Japan, the incidence of SMA is approximately 1 in 20,000 live births [6]. SMA can be classified into subtypes based on age of onset and disease severity (i.e., the maximum achieved motor milestones) [1]. Type 1 is the most severe type of SMA, in which the patients have an onset of clinical signs before the age of 6 months and require ventilation. Type 2 is characterized by age of onset before the age of 18 months; patients can sit unsupported but do not have the ability to stand or walk unsupported. Type 3 includes clinically heterogenous patients who have an onset of disease after the age of 18 months; disease progresses with patients losing their ability to walk during the course of the disease. Type 4 is an adult-onset SMA, manifesting after the age of > 30 years [7].

Genetically, most patients with SMA have a homozygous gene deletion or loss-of-function mutation in the *survival motor neuron 1* (*SMN1*) gene on chromosome 5q13 (5q SMA) [7]. A paralogous gene, *SMN2,* is highly homologous to *SMN1* and lies within the same chromosome region 5q13 [1, 7]. Both *SMN1* and *SMN2* encode the SMN protein; however, the *SMN2* gene has a nucleotide difference of 840C>T in exon 7 that affects the splicing pattern [7, 8]. This results in a transcript missing exon 7, which then encodes a non-functioning SMN protein. *SMN2* is considered a modifying factor of disease severity, with a greater copy number of *SMN2* associated with a milder form of SMA [1]. Most individuals affected with SMA have a homozygous deletion of *SMN1* exon 7 or a gene conversion from *SMN1* to *SMN2* [7]. However, other intragenic variants, including missense, nonsense, and splice site mutations, can also occur in any of the other *SMN1* exons [1].

With the availability of disease-modifying therapy for SMA in recent years, genetic testing has become increasingly important. The multiplex ligation-dependent probe amplification (MLPA) method is a clinically available genetic test used for the genetic diagnosis of 5q SMA [9]. However, this method only detects copy number variants of *SMN1* and is unable to capture intragenic variants within exons and introns. Thus, patients with clinically diagnosed SMA may not be genetically diagnosed with SMA using this method. To overcome this limitation of the MLPA method, a method using long-range polymerase chain reaction (LR-PCR) for *SMN* gene amplification was developed [10]. The LR-PCR method enabled the detection of intragenic variants within *SMN1* (exons 1–8) and hybrid *SMN* genes where *SMN2* exon 7 recombines with *SMN1* exon 8. The identification of single-nucleotide variants (SNVs) for each exon/intron of *SMN1* and hybrid SMN genes may help guide the diagnosis and treatment of 5q SMA.

The aim of this study was to analyze all exons and introns of *SMN1* in Japanese patients with clinically diagnosed SMA to make a genetic diagnosis of SMA, and to identify SNVs in *SMN1* that may have a clinical impact. To facilitate our analysis, we modified the primers of the LR-PCR method to enable next-generation sequencing (NGS) of all exon and intron regions of *SMN1.*

## Materials and methods

### Study design

This was a retrospective observational study using pre-collected DNA samples from patients at Tokyo Women’s Medical University with clinically diagnosed SMA. Patient DNA samples were collected between January 2017 and March 2019. Patients were enrolled for this study between January 28, 2020 and September 30, 2021. Written informed consent was obtained from each patient or their legal representative prior to data collection, and patient data collected were de-identified and anonymized. A motor function questionnaire (Additional file 1) was sent to attending physicians of patients who provided written informed consent to assess patient’s maximum and current motor function. The study was conducted in accordance with the protocol, ethical principles of the Declaration of Helsinki, and all relevant regulations and guidelines. The study protocol and the informed consent were approved on September 17, 2019 by the Ethics Committee of the Tokyo Women’s Medical University (approval no. 393B). This study was registered with the University Hospital Medical Information Network Clinical Trials Registry (registration number: UMIN000040095).

### Patient DNA samples

Genomic DNA patient samples were those from patients who had consented to participate in this study and had a clinically confirmed diagnosis of SMA. DNA samples with associated patient demographic and clinical data, including age at onset, SMA type, and maximum and current motor function, were included. Genomic DNA patient samples without the associated patient demographic and clinical data available were excluded.

**Fig. 1:**
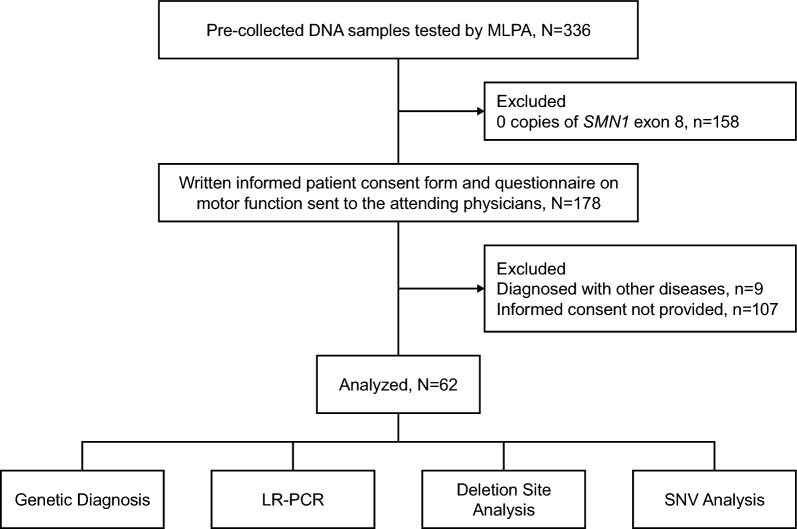
Patient DNA sample flow. LR-PCR, long-range polymerase chain reaction; MLPA, multiplex ligation-dependent probe amplification; *SMN1*, survival motor neuron 1; SNV, single nucleotide variant

From an initial 336 DNA samples stored at the Tokyo Women’s Medical University from patients with clinically diagnosed SMA, 158 DNA samples were identified with 0 copies of *SMN1* exon 8 and were excluded from this study because such samples are not amenable to the LR-PCR method used (Fig. 1). From the remaining 178 patient DNA samples, 62 patient samples were included in this study; 107 patients were excluded due to not providing their informed consent and nine patients were diagnosed with other diseases.

### Genetic analysis

#### Analysis of SMN copy number

Pre-collected genomic DNA samples were thawed at room temperature. DNA samples had been extracted from peripheral blood leukocytes either manually using the QIAamp DNA Blood Mini Kit (Qiagen, Hilden, Germany) or by automated extraction with the Maxwell and Maxwell RSC Blood DNA Kit (Promega Corporation, Mannheim, Germany; Promega Corporation, Madison, USA). As a preliminary analysis to detect copy number and to identify deletions within the *SMN1* and *SMN2* genes, MLPA was performed using SALSA MLPA Probemix P021-B1 SMA (MRC-Holland, Amsterdam, Netherlands) as previously described [10].

#### Mutational analysis

For specific amplification of *SMN1*, LR-PCR, which has been described previously [10] and modified for this study, was performed using KOD FX Neo polymerase (TOYOBO, Osaka, Japan). Forward primer hybridization, 654 bp from the transcription initiation site, was performed using modified forward and reverse primers (Table 1) to amplify a 28.2-kb region that included *SMN1* exons 1–8 as described previously [10]. After amplification, the LR-PCR 28.2-kb product was confirmed by 1.0% agarose gel electrophoresis. Each amplified *SMN1* product was excised from the agarose gel and extracted using the QIAEX II Gel Extraction Kit (Qiagen, Hilden, Germany). Analysis of intragenic SNVs and deletions and hybrid *SMN* genes was conducted on the purified LR-PCR products by NGS (MiSeq^®^, Illumina, Inc., San Diego, CA, USA) according to the manufacturer’s instructions. The method for converting the long PCR product to NGS-compatible short fragments was paired-end 150 bp (Read Type: "Paired End"; Cycles Read 1: "151", Cycles Read 2: "151") using the Nextera XT DNA Library Preparation Kit (Illumina, Inc., San Diego, CA, USA). For variant annotation and filtering, the default criteria of the Illumina VariantStudio v3.0 were used, and data were analyzed using Software Version 3.0.12 (Illumina, Inc., San Diego, CA, USA) and Integrative Genomics Viewer (IGV) [11]. The default transcript for *SMN1* was NM_000344. All coordinates for SNVs and mutations identified in this study are based on the human genome build GRCh37/hg19. P-values were determined by Fisher's exact test performed using GraphPad Prism version 10.2.3 (GraphPad Software, Boston, MA, USA).

**Table 1:** Modified primer design for amplification of *SMN1*

Methods	Forward primer	Reverse primer	Product length Transcript position
Original method^a^	GTTGGGGGATCAAATATCTTCTAGTGTT	CCCCCACCCCAGTCTTTTACAGATGGT	28,233 bp Hg19 chr5: 70,220,277~70,248,509
Modified method	CCCATGTTTGTCTTTCCTTGTTTGTCT	CCACCCCAGTCTTTTACAGATGGTTT	28,175 bp Hg19 chr5: 70,220,332~70,248,506

*bp,* base pairs*; chr,* chromosome; *hg,* human genome; *SMN1*, survival motor neuron 1

^a^As previously described [10]

## Results

### Patient characteristics

The patient cohort consisted of both adult and pediatric patients with SMA types 1, 2, 3, and 4 (Table 2). For half the patient cohort, the maximum motor function was climbing up stairs. Tracheostomy was reported for six patients. Seven (11.3%) patients had 0 copies of *SMN1* exon 7, and 7 (11.3%) patients had one copy of *SMN1* exon 7. For one patient, there were 0 copies of *SMN2* exon 7 and exon 8*.*

**Table 2:** Patient clinical characteristics and *SMN* copy number information (N = 62)

Characteristic	SMA type^a^				Total N = 62
	1 (n = 9)	2 (n = 13)	3 (n = 24)	4 (n = 16)	
Age of onset					
≤ 2 years	4 (44.4)	5 (38.5)	7 (29.2)	0 (0)	16 (25.8)
3–10	0 (0)	0 (0)	4 (16.7)	0 (0)	4 (6.5)
11–20	0 (0)	0 (0)	0 (0)	1 (6.3)	1 (1.6)
21–30	0 (0)	0 (0)	0 (0)	1 (6.3)	1 (1.6)
31–40	0 (0)	0 (0)	0 (0)	3 (18.8)	3 (4.8)
41–50	0 (0)	0 (0)	0 (0)	2 (12.5)	2 (3.2)
≥ 51	0 (0)	0 (0)	0 (0)	5 (31.3)	5 (8.1)
Unknown	5 (55.6)	8 (61.5)	13 (54.2)	4 (25.0)	30 (48.4)
*SMN1* exon 7 copy number					
0 copies	2 (22.2)	2 (15.4)	3 (12.5)	0 (0)	7 (11.3)
≥ 1 copy	7 (77.8)	11 (84.6)	21 (87.5)	16 (100)	55 (88.7)
Maximum motor function					
Climbing up stairs	0 (0)	0 (0)	17 (70.8)	15 (93.8)	32 (51.6)
Walking independently	0 (0)	0 (0)	7 (29.2)	1 (6.3)	8 (12.9)
Walking with assistance	0 (0)	6 (46.2)	0 (0)	0 (0)	6 (9.7)
Stands with assistance	0 (0)	0 (0)	0 (0)	0 (0)	0 (0)
Shuffling in sitting position	0 (0)	6 (46.2)	0 (0)	0 (0)	6 (9.7)
Pivots (rotate) (on the spot)	0 (0)	0 (0)	0 (0)	0 (0)	0 (0)
Sitting independently	0 (0)	1 (7.7)	0 (0)	0 (0)	1 (1.6)
Head upright all the time	5 (55.6)	0 (0)	0 (0)	0 (0)	5 (8.1)
Unable to maintain head upright	4 (44.4)	0 (0)	0 (0)	0 (0)	4 (6.5)
Current motor function					
Climbing up stairs	0 (0)	0 (0)	10 (41.7)	6 (37.5)	16 (25.8)
Walking independently	0 (0)	0 (0)	5 (20.8)	5 (31.3)	10 (16.1)
Walking with assistance	0 (0)	4 (30.8)	3 (12.5)	1 (6.3)	8 (12.9)
Stands with assistance	0 (0)	2 (15.4)	3 (12.5)	1 (6.3)	6 (9.7)
Shuffling in sitting position	0 (0)	5 (38.5)	1 (4.2)	1 (6.3)	7 (11.3)
Pivots (rotate) (on the spot)	0 (0)	0 (0)	1 (4.2)	0 (0)	1 (1.6)
Sitting independently	0 (0)	0 (0)	0 (0)	1 (6.3)	1 (1.6)
Head upright all the time	4 (44.4)	2 (15.4)	1 (4.2)	1 (6.3)	8 (12.9)
Unable to maintain head upright	4 (44.4)	0 (0)	0 (0)	0 (0)	4 (6.5)
Unknown	1 (11.1)	0 (0)	0 (0)	0 (0)	1 (1.6)
Tracheostomy					
Yes	3 (33.3)	1 (7.7)	1 (4.2)	1 (6.3)	6 (9.7)
No	5 (55.6)	12 (92.3)	23 (95.8)	15 (93.8)	55 (88.7)
Unknown	1 (11.1)	0 (0)	0 (0)	0 (0)	1 (1.6)
*SMN1*					
Exon 7 copy number					
0	2 (22.2)	2 (15.4)	3 (12.5)	0 (0)	7 (11.3)
1	3 (33.3)	0 (0)	4 (16.7)	0 (0)	7 (11.3)
2	3 (33.3)	10 (76.9)	17 (70.8)	15 (93.8)	45 (72.6)
3	1 (11.1)	1 (7.7)	0 (0)	1 (6.3)	3 (4.8)
Exon 8 copy number					
1	5 (55.6)	2 (15.4)	6 (25.0)	0 (0)	13 (21.0)
2	3 (33.3)	10 (76.9)	18 (75.0)	14 (87.5)	45 (72.6)
3	1 (11.1)	1 (7.7)	0 (0)	2 (12.5)	4 (6.5)
*SMN2*					
Exon 7 copy number					
0	0 (0)	0 (0)	1 (4.2)	0 (0)	1 (1.6)
1	2 (22.2)	4 (30.8)	11 (45.8)	9 (56.3)	26 (41.9)
2	6 (66.7)	8 (61.5)	9 (37.5)	7 (43.8)	30 (48.4)
3	1 (11.1)	1 (7.7)	2 (8.3)	0 (0)	4 (6.5)
4	0 (0)	0 (0)	1 (4.2)	0 (0)	1 (1.6)
Exon 8 copy number					
0	0 (0)	0 (0)	1 (4.2)	0 (0)	1 (1.6)
1	2 (22.2)	4 (30.8)	13 (54.2)	10 (62.5)	29 (46.8)
2	6 (66.7)	9 (69.2)	9 (37.5)	6 (37.5)	30 (48.4)
3	1 (11.1)	0 (0)	0 (0)	0 (0)	1 (1.6)
4	0 (0)	0 (0)	1 (4.2)	0 (0)	1 (1.6)

SMA, spinal muscular atrophy; *SMN1*, survival motor neuron 1; *SMN2*, survival motor neuron 2

Data are n (%)

^a^In patients with ≥ 1 copy of *SMN1* exon 7, non-5q SMA that were not caused by *SMN1* were included

### Genetic diagnosis of SMA

**Fig. 2:**
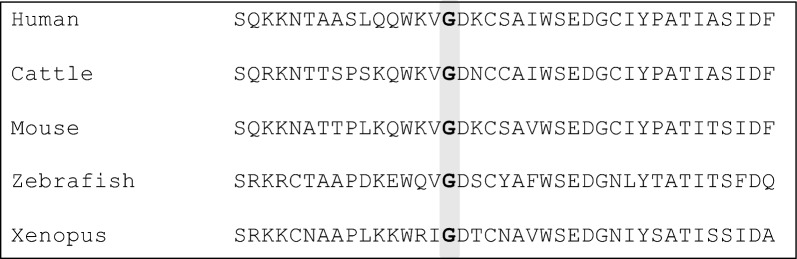
Amino acid alignment of exon 3 of *SMN1.* The location of the conserved glycine that is changed in the p.G95E homozygous missense mutation is shown in bold text. Multiple sequence alignment was performed using CLUSTALW (10 May 2022). *SMN1*, survival motor neuron 1

After *SMN* copy number analysis using MLPA, sequencing analysis was performed on the 62 patient samples. Two female patients had pathogenic mutations and were genetically diagnosed with SMA (Table 3). One female patient was 9 years of age who had a tracheostomy at 1 year of age, and gastrostomy and fundoplication at 3 years of age. Copy number variations for this patient were: *SMN1,* exon 7 one copy, exon 8 one copy; *SMN2,* exon 7 two copies, exon 8 two copies. Sequencing analysis identified a heterozygous mutation in intron 6 of *SMN1* (c.835-3C>A); this mutation had been previously reported [12]. The second female patient was 56 years of age, with a history of muscle weakness of the left foot at age 12 years, who required a walking cane at age 30 years and a wheelchair at age 45 years; the patient had a family history (first-cousin marriage) of disease. Copy number variations for this patient were: *SMN1,* exon 7 two copies, exon 8 two copies; *SMN2,* exon 7 two copies, exon 8 two copies. Sequencing analysis identified a novel homozygous missense mutation in exon 3 (c.284G>A; p.G95E) at a site where glycine is typically conserved across species (Fig. 2).

**Table 3:** Sequencing analysis of *SMN1* for two patients with a genetic diagnosis of SMA

Patient	Variant location	Zygosity	Nucleotide change	CADD score	SIFT	Poly-Phen2	MaxEntScan	TogoVar	Functional studies	ACMG guideline
1	Intron 6	Heterozygous (*SMN1*, 1 copy)	c.835–3 C>A	22.2	‒	‒	8.26^a^	rs772466166	Wijaya et al. (2021) reported a defect in exon 7 splicing, identified by *SMN* transcript analysis	Category PS3^b^, PM2^c^, PM3^d^; likely pathogenic (criteria ii)
2	Exon 3	Homozygous (*SMN1*, 2 copies)	c.284 G>A	24.4	Deleterious	Probably damaging	‒	‒	‒	Category PM1^e^, PM2^c^, PM5^f^; likely pathogenic (criteria iv)

ACMG, American College of Medical Genetics; CADD, Combined Annotation Dependent Depletion; Poly-Phen2, Polymorphism Phenotyping v2; PM, pathogenic moderate; PS, pathogenic strong; SMN1, survival motor neuron 1; SIFT, Sorting Intolerant from Tolerant

^a^Reported in Wijaya et al. (2021)

^b^The functional study reported in Wijaya et al. (2021) shows this variant has a deleterious effect

^c^The allele frequencies of these variants have not been reported in any control population

^d^One allele is deleted and there is a point mutation in the other allele

^e^The location of this variant is in the highly conserved Tudor domain of SMN (amino acids 92–144; Sun et al. [2005])

^f^An alternative pathogenic variant at this location (p.G95R) was previously identified in a patient with type 3 SMA, as described in Sun et al. (2005)

### Modified LR-PCR amplification method

**Fig. 3:**
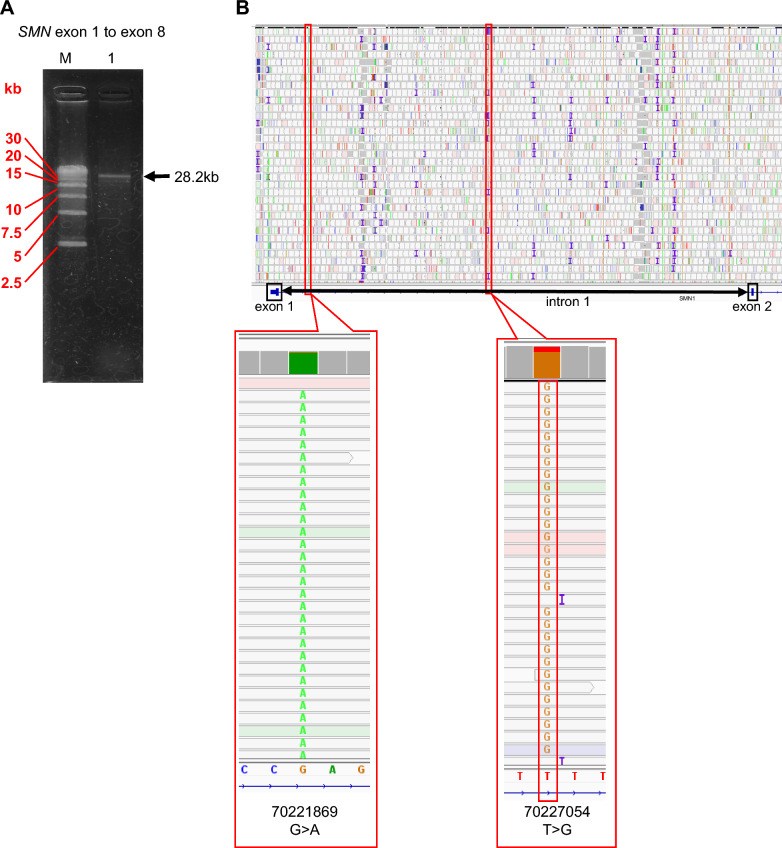
Evaluation of the modified LR-PCR method. *SMN1*-specific LR-PCR amplifications are shown. Patient sample consisted of two copies of *SMN1* and two copies of *SMN2*. **A** Amplification from exon 1 to exon 8 is shown; in addition to the 28.2 kb target band, there are also non-specific bands. Lane M. Molecular weight marker, Lane 1. Modified method. **B** Increased coverage of exon 1 to intron 1 of *SMN1* with the modified LR-PCR method. Two SNVs, and corresponding nucleotide numbers, in intron 1 of *SMN1* identified using the modified LR-PCR method are shown. LR-PCR, long-range polymerase chain reaction; *SMN1*, survival motor neuron 1; *SMN2*, survival motor neuron 2; SNVs, single nucleotide variants

For specific amplification of *SMN1*, LR-PCR, which has been described previously [10], was modified for this study. LR-PCR was performed on 13 DNA samples and a 28.2-kb region that included *SMN1* exons 1–8 was amplified. The target band was identified in addition to non-specific bands (Fig. 3A). NGS analysis of the extracted 28.2 kb target band revealed good coverage from exon 1 to intron 1 of *SMN1* (Fig. 3B) and previously unknown SNVs in intron 1 were detected (Additional file 2).

### *SMN1* SNV analysis

**Fig. 4:**
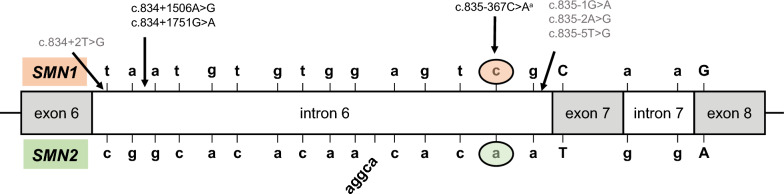
Single nucleotide variants identified in *SMN1* intron 6. *SMN1* intron 6 single nucleotide variants identified in this study are shown in black font. Previously reported *SMN1* intron 6 pathogenic mutations are shown in grey font [1, 17, 18]. ^a^Identified in this study and previously reported as a SNV [17]. *SMN1*, survival motor neuron 1; *SMN2*, survival motor neuron 2; SNV, single nucleotide variant

SNV analysis on the genomic sequence of *SMN1* was performed. Among the 62 patient samples analyzed, variants of interest that were identified included 3 SNVs in intron 6 (Table 4 and Fig. 4): c.835-367C>A (n = 5), c.834+1751G>A (n = 2), c.834+1506A>G (n = 9). Variants within intron 6 of *SMN1* were identified at a numerically higher frequency in patients with 0 copies of *SMN1* exon 7 compared with patients with ≥ 1 copy of *SMN1* exon 7 and was statistically significantly higher for the c.835-367C>A and c.834+1506A>G variants (Table 4).

**Table 4:** *SMN1* intron 6 single nucleotide variants of interest

Intron 6 variant	*SMN1* exon 7 copy number					*SMN1* copy number		*SMN2* copy number	
	0 copies (N = 7) n (%)	≥ 1 copy (N = 55) n (%)	*P* value^a^	0 copies Patient ID	SMA type	Exon 7	Exon 8	Exon 7	Exon 8
c.835-367C>A	5 (71.4)	0 (0)	< 0.0001	A	3	0	2	3	1
				B	3	0	1	2	1
				C	1	0	1	2	2
				D	2	0	1	2	2
				E	1	0	1	2	2
c.834+1751G>A	1 (14.3)	1 (1.8)	0.2147	B	3	0	1	2	1
c.834+1506A>G	4 (57.1)	5 (9.1)	0.0064	A	3	0	2	3	1
				B	3	0	1	2	1
				C	1	0	1	2	2
				E	1	0	1	2	2

ID, identifier; SMA, spinal muscular atrophy; *SMN1*, survival motor neuron 1; *SMN2*, survival motor neuron 2

^a^*P* value between 0 copies versus ≥ 1 copy

### Hybrid SMN analysis

**Fig. 5:**
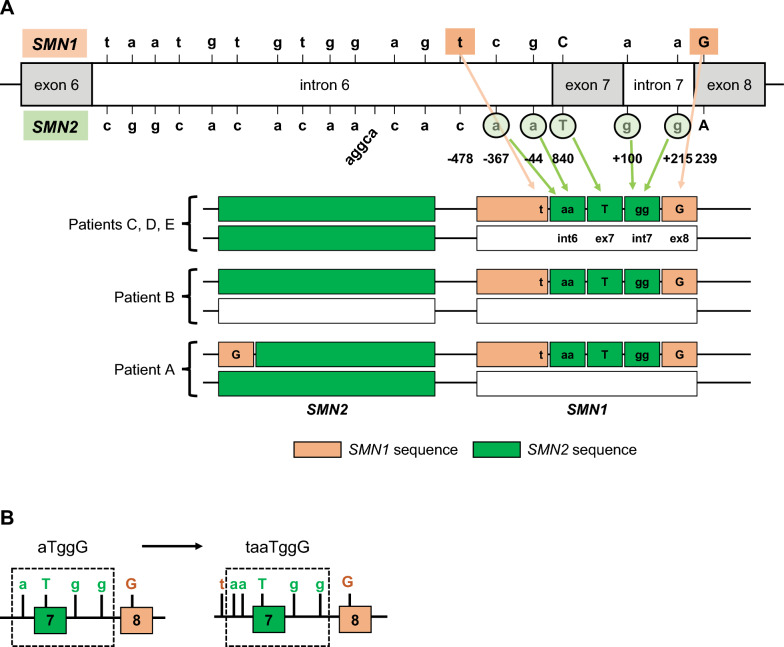
Schematic illustration of the hybrid *SMN* gene types. **A** Genomic structure of normal *SMN* genes and three types of genomic structures of the hybrid *SMN* genes identified in this study. **B** *SMN1*-to-*SMN2* gene conversion. Dotted line frames *SMN2* sequence. *SMN1*, survival motor neuron 1; *SMN2*, survival motor neuron 2

Five patients with a homozygous deletion of *SMN1* exon 7 but not exon 8 (A, B, C, D, E; Table 4) were examined for the hybrid *SMN* gene. LR-PCR of a region that included exons 1–8 of *SMN1* was performed, followed by sequencing of intron 6, exon 7, intron 7, and exon 8. We confirmed patterns of the hybrid *SMN* gene type (Fig. 5A) and the sequence of the hybrid *SMN* intron 6, exon 7, intron 7, and exon 8 was taaTggG (Fig. 5B).

## Discussion

In this study, the intron and exon regions of *SMN1* were examined for SNVs and hybrid *SMN* genes in Japanese patients with clinically diagnosed SMA for whom no *SMN* copy number variation was identified by the standard MLPA method. By NGS, point mutations were identified in *SMN1* and a genetic diagnosis of SMA was made for two patients. For the remaining patients, deep intronic variants were detected in intron 6 more frequently in patients with 0 copies of *SMN1* exon 7 than in patients with ≥ 1 copies of *SMN1* exon7. A new hybrid *SMN* gene was identified in five patients. These results highlight that the current MLPA method used for the genetic diagnosis of SMA does not detect all *SMN* gene variants and that additional methods are required for the accurate genetic diagnosis of SMA.

Vezain et al. [13] have previously shown that the *SMN1* c.835-3C>T mutation, identified in a patient with type 4 SMA diagnosed at age 44 years, induces a defect in the splicing of exon 7, resulting in the exclusion of exon 7 from *SMN1* transcripts. In this study, we identified a heterozygous mutation (c.835-3C>A) at the same location of intron 6 of *SMN1* and hypothesized that the c.835-3C>A mutation would also modify the splicing of *SMN1* exon 7. At the time of this study, the c.835-3C>A intragenic mutation was identified in a Japanese patient with SMA type 1 (*SMN1,* exon 7 one copy, exon 8 one copy) with a severe phenotype despite having two copies of *SMN2* [12]; SMN transcript analysis showed that *SMN1* exon 7 was deleted, indicating that the c.835-3C>A intragenic mutation does affect the splicing of exon 7. Furthermore, the mutation is deemed “likely pathogenic” based on the American College of Medical Genetics (ACMG) criteria [14]. In this study, for the remaining six patients with one copy of *SMN1*, no pathogenic variant could be detected within *SMN1* and further investigation of *SMN1* and surrounding genes may be required. In a second patient, we identified a homozygous point mutation (c.284G>A) that resulted in a change of the conserved amino acid glycine to glutamic acid (p.G95E); the mutation was homozygous because this family had a consanguine marriage (first-cousin marriage). A previous study has shown via an in vitro assay that the missense mutation p.G95R reduces the ability of the SMN protein to bind to spliceosomal (Sm) proteins [15]. The investigators assumed this disrupted binding of SMN to Sm proteins would lead to insufficient uridine-rich small nuclear ribonucleoprotein biogenesis, which is essential for RNA splicing [16]. We therefore concluded that the p.G95E missense mutation would also reduce the ability of SMN to bind to Sm proteins, and thus is a pathogenic mutation. This conclusion is supported by the p.G95E mutation meeting the ACMG criteria for pathogenicity, a Combined Annotation Dependent Depletion score of 24.4, a Sorting Intolerant From Tolerant classification of “deleterious”, and a Polymorphism Phenotyping v2 classification of “probably damaging” [14]. Furthermore, the patient with the c.284G>A mutation (p.G95E) in this study eventually received treatment for SMA.

In the current study, the previously described LR-PCR method [10] was modified to enable NGS analysis of exons 1–8 and the intron 1/exon 1 region of *SMN1*. We identified SNVs in the intron 1 region, which have not been reported previously, and 3 SNVs in deep intron 6 of *SMN1* in patients with SMA. The c.834+1506A>G and c.834+1751G>A variants were identified in patients with 0 copies of *SMN1* exon 7 and patients with ≥ 1 copies of *SMN1* exon 7; the remaining SNV (c.835-367C>A) was identified only in patients with 0 copies of *SMN1* exon 7. SNVs within intron 6 have been reported previously including c.835-367C>A, some of which were positioned close to the splice sites of exon 6 and exon 7 (Fig. 4) [1, 12, 17, 18]. Ruhno et al. [18] reported 3 SNVs in intron 6, which were significantly more common in patients with a milder disease severity than was expected from their *SMN2* copy number. In the present study, SNVs in intron 6 were identified in five patients with 0 copies of *SMN1* exon 7 and variable phenotypes. Two patients (C and E) had a severe phenotype, with two copies of *SMN2* exon 7 and exon 8; both were positive for c.834+1506A>G and c.835-367C>A in intron 6. Patient C could keep their head upright all the time, whereas Patient E was unable to maintain their head upright. Patients A and B were also positive for c.834+1506A>G and c.835-367C>A, but their disease was less severe. Patient A had three copies of *SMN2* exon 7 and 1 copy of *SMN2* exon 8, and was capable of standing with assistance. Patient B had two copies of *SMN2* exon 7 and 1 copy of *SMN2* exon 8, and could climb up stairs. Patient B was also positive for a third SNV in intron 6 (c.834+1751G>A). Patient D was positive for only one SNV in intron 6 (c.835-367C>A) and had two copies of *SMN2* exon 7 and exon 8. Their disease was mild, they had the ability to stand with assistance, and they were previously capable of walking with assistance. Given the varied phenotypes observed in these patients, these variants and those that occurred more frequently in patients with 0 copies of *SMN1* exon 7 than in patients with ≥ 1 copies of *SMN1* exon 7 in our present research will be examined in the future with consideration of the possibility of disease-modifying factors.

Hybrid *SMN* genes have been identified in patients with SMA from various ethnic groups [10, 19–24]. The most common *SMN* gene sequence is aTggG, but aTgaG, aTaaG, aTagG, gTaaG, and aTaga sequences have also been reported [10, 19, 23, 24]. In this current study, a new hybrid *SMN* gene sequence was identified (taaTggG) that consisted of upstream “t” and downstream “G” sequences of *SMN1* and “aaTgg” sequence of *SMN2*. The diversity of hybrid *SMN* gene sequences observed in patients with SMA may be explained in part by the instability of the downstream sequence of the *SMN* gene. More than 60 Alu-like sequences occupy approximately 41% of the human *SMN* gene, including the promoter region [25]. Alu elements are an abundant short, interspersed repetitive element in the human genome consisting of approximately 300 base pairs in length (for a review, see article by Deininger P [26]). Alu elements can affect posttranscriptional processes such as pre-messenger RNA (mRNA) splicing and mRNA stability [27]. An Alu-rich region is present in intron 6 of the *SMN* gene, which may be involved in the deletion of exon 7 and 8 [25]. The existence of such repetitive downstream sequences of the *SMN* gene may be the cause of the diversity in the sequence of the hybrid *SMN* genes observed in patients with SMA. *SMN1* and *SMN2* copy number may have different gene origins or sequences due to the existence of the hybrid gene and gene conversion. It is therefore hoped that the future elucidation of genotype–phenotype correlation will improve the accuracy of predicting disease severity and treatment responses by copy number, and for this, full-length sequencing will become increasingly important in the diagnosis of SMA.

The strengths of this study were that more than 60 Japanese patients with clinically diagnosed SMA were included in the SNV analysis, the patient population consisted of both pediatric and adult patients with clinically diagnosed SMA, SMA types 1–4 were included, and the full genomic sequence of *SMN1* was analyzed using NGS. The limitation of this study was that due to the design of the primers for *SMN1* exon 8, cases with *SMN1* exon 8 deleted were excluded from the analysis, and only those with exon 7 deleted, i.e., mild cases, could be analyzed. Lastly, the study cohort was small and consisted of only Japanese patients; therefore, additional studies are required to confirm if these results can be generalized to other populations.

## Conclusions

In this study of Japanese patients with clinically diagnosed SMA, deep intronic SNVs in intron 6 were identified more frequently in patients with 0 copies of *SMN1* exon 7 than in patients with ≥ 1 copies of *SMN1* exon 7. In addition, we identified in five patients a hybrid *SMN* gene that contained the *SMN2* sequence “aaTgg” flanked by an upstream “t” and downstream “G” sequence of *SMN1*. Furthermore, the two cases that were genetically diagnosed with SMA are those that are not detected through the current genetic screening for newborns. In the era of expanded newborn screening, it will be increasingly important to develop *SMN* sequencing methods that can complement current screening by MLPA methods.

## Supplementary Information

Additional file1.

## Data Availability

The datasets used and analyzed during the current study are not openly available due to reasons of sensitivity and are available from the corresponding author on reasonable request. Data are located in controlled access data storage at Tokyo Women's Medical University. Variants identified in the study are planned to be registered in the Leiden Open Variation Database (https://databases.lovd.nl/shared/genes).

## Abbreviations

LR-PCR: Long-range polymerase chain reaction
MLPA: Multiplex ligation-dependent probe amplification
mRNA: Messenger RNA
NGS: Next-generation sequencing
Sm: Spliceosomal
SMA: Spinal muscular atrophy
SMN1: Survival motor neuron 1
SMN2: Survival motor neuron 2
SNV: Single nucleotide variant
