# Supplementary material for: Genomic analysis of the SMN1 gene region in patients with clinically diagnosed spinal muscular atrophy: a retrospective observational study
Source: Orphanet J Rare Dis. 2025 Feb 7;20:55. doi: 10.1186/s13023-025-03568-9 (PMC11803984; doi:10.1186/s13023-025-03568-9)
Supplement: Supplementary file 1 — Additional file1. [file 13023_2025_3568_MOESM1_ESM.docx]

SUPPLEMENTARY MATERIAL

**TITLE**

**Genomic analysis of the *SMN1* gene region in patients with clinically diagnosed spinal muscular atrophy: a retrospective observational study**

Tamaki KATO^1^, Mamoru YOKOMURA^1^, Yutaka OSAWA^2^, Kensuke MATSUO^3^, Yuji KUBO^1^,

Taihei HOMMA^4^, Kayoko SAITO^1^

AUTHOR AFFILIATIONS

^1^ Institute of Medical Genetics, Tokyo Women’s Medical University, Tokyo, Japan

^2^ Department of Neurology, Kawasaki Medical School, Kurashiki, Okayama, Japan

^3^ Division of Pediatrics, Kyoto Tanabe Central Hospital, Kyoto, Japan

^4^ Biogen Japan Ltd., Tokyo, Japan

AUTHOR FOR CORRESPONDENCE

Kayoko Saito

Institute of Medical Genetics

Tokyo Women’s Medical University

8-1 Kawada-cho, Shinjuku-ku

Tokyo 162-8666

Japan

Tel: +81-3-3353-8111

Email: saito.kayoko@twmu.ac.jp

**LIST OF TABLES AND FIGURES**

**Additional file 1** Motor function questionnaire

**Additional file 2** Single nucleotide variants in intron 1 of *SMN1*

**Additional file 1** Motor function questionnaire

| **Questionnaire at the time of obtaining consent for research on the relationship between genotype and phenotype by genomic analysis of causative gene regions  in SMA** | | | |
| --- | --- | --- | --- |
| Thank you for agreeing to the research above.  To begin this research, we would appreciate your cooperation in completing  the following questionnaire.  The information provided in the questionnaire will be used only for this research  and will be handled in strict confidence. | | | |
| Research ID no. |  |  |  |
|  |  |  |  |
| 1. Date of birth* | Year: | Month: | Date: |
| *From a personal information point of view, you can provide the information up to "month" | | | |
| 2. Treatment history of SPINRAZA^®^ | 1: Yes | 2: No | 3: Unknown |
| 2.1 If "yes", the presence or absence of treatment effects | 1: Yes  2: No |  |  |
| 3. History of valproic acid use | 1: Yes | 2: No | 3: Unknown |
| 4. Maximum motor function | Age at achievement: | Year: | Month: |
|  | 1. Climbing up stairs | 6. Pivots (rotate)  (on the spot) |  |
|  | 2. Walking independently | 7. Sitting independently |  |
|  | 3. Walking with assistance | 8. Head upright  all the time |  |
|  | 4. Stands with assistance | 9. Unable to maintain head upright |  |
|  | 5. Shuffling in sitting position |  |  |
| 5. Current motor function | Age at achievement: | Year: | Month: |
|  | 1. Climbing up stairs | 6. Pivots (rotate)  (on the spot) |  |
|  | 2. Walking independently | 7. Sitting independently |  |
|  | 3. Walking with assistance | 8. Head upright  all the time |  |
|  | 4. Stands with assistance | 9. Unable to maintain head upright |  |
|  | 5. Shuffling in sitting position |  |  |
| 6. Presence or absence of ventilatory therapy under tracheostomy ventilation | 1: Present | 2: Absent |  |
|  |  |  |  |
| 7. Request for disclosure of  research results | 1: Yes | 2: No |  |
| This is the end of the survey. Thank you very much for your cooperation. | | | |
| Please return this along with the consent form using the return envelope. | | | |

**Additional file 2** Single nucleotide variants in intron 1 of *SMN1* identified using the modified LR-PCR method (N=13)

| ***SMN1* nucleotide number** | **Base change** | **n (%)** |
| --- | --- | --- |
| 70221869 | G>A | 1 (7.7) |
| 70222648 | G>A | 1 (7.7) |
| 70225245 | T>C | 4 (30.8) |
| 70226900 | A>C | 4 (30.8) |
| 70227054 | T>G | 9 (69.2) |
| 70227064 | T>G | 4 (30.8) |
| 70227569 | A>T | 3 (23.1) |
| 70229093 | T>C | 3 (23.1) |

LR-PCR, long-range polymerase chain reaction; *SMN1*, survival motor neuron
